# Transient Stroke-Like Symptoms Following Subcutaneous Lidocaine Administration: A Case Report

**DOI:** 10.7759/cureus.99780

**Published:** 2025-12-21

**Authors:** Naomi R Khanna, Adil Pervaiz

**Affiliations:** 1 Cardiology, Advanced Cardiology LLC, Hackettstown, USA; 2 Biomedical Engineering, Columbia University, New York City, USA; 3 Cardiology, Morristown Medical Center, Morristown, USA

**Keywords:** lidocaine, local anesthesia, local anesthetic systemic toxicity, stroke-like symptoms, subcutaneous injection, transient neurological symptoms

## Abstract

Transient neurological symptoms can occasionally occur as a complication of procedures involving the use of the local anesthetic lidocaine. The present report outlines a case of a patient who developed acute but self-limited neurological symptoms following subcutaneous lidocaine administration for a minimally invasive outpatient procedure. Shortly after receiving 20 cc of lidocaine, she experienced slurred speech that resolved within 30 minutes. Neuroimaging did not reveal any evidence of acute pathology. This case highlights a rare instance of transient neurological symptoms following lidocaine administration, underscoring the need for clinician vigilance and preventive strategies during local anesthetic use.

## Introduction

Lidocaine is routinely employed as a local anesthetic, with common use in outpatient procedural settings due to its efficacy and reasonable safety profile [1-3]. Lidocaine-related complications, especially neurological complications, are rare [1,4]. The overall incidence is less than 3% [4], and most reported events involve effects on the nerve supply at the injection site. The incidence of true stroke-like symptoms is not well known and remains poorly described. This case report details the immediate onset of transient neurological symptoms following subcutaneous lidocaine administration, in which the initial working diagnosis was a stroke or transient ischemic attack (TIA).

Given the widespread use of lidocaine as a local anesthetic, the purpose of presenting this case is to raise clinicians’ awareness of potential complications such as stroke-like symptoms and to highlight preventive strategies discussed later in this report.

## Case presentation

An 86-year-old woman with a history of recurrent palpitations, Mobitz type I heart block, and supraventricular tachycardia (SVT) recorded on ambulatory Holter monitoring underwent an in-office implantation of an implantable loop recorder (ILR) (Boston Scientific, USA). The procedure was performed under local anesthesia with 20 cc of 1% lidocaine (without epinephrine) administered subcutaneously at the site of incision. Shortly after the procedure, the patient experienced difficulty speaking and was taken to the hospital emergently.

**Figure 1:**
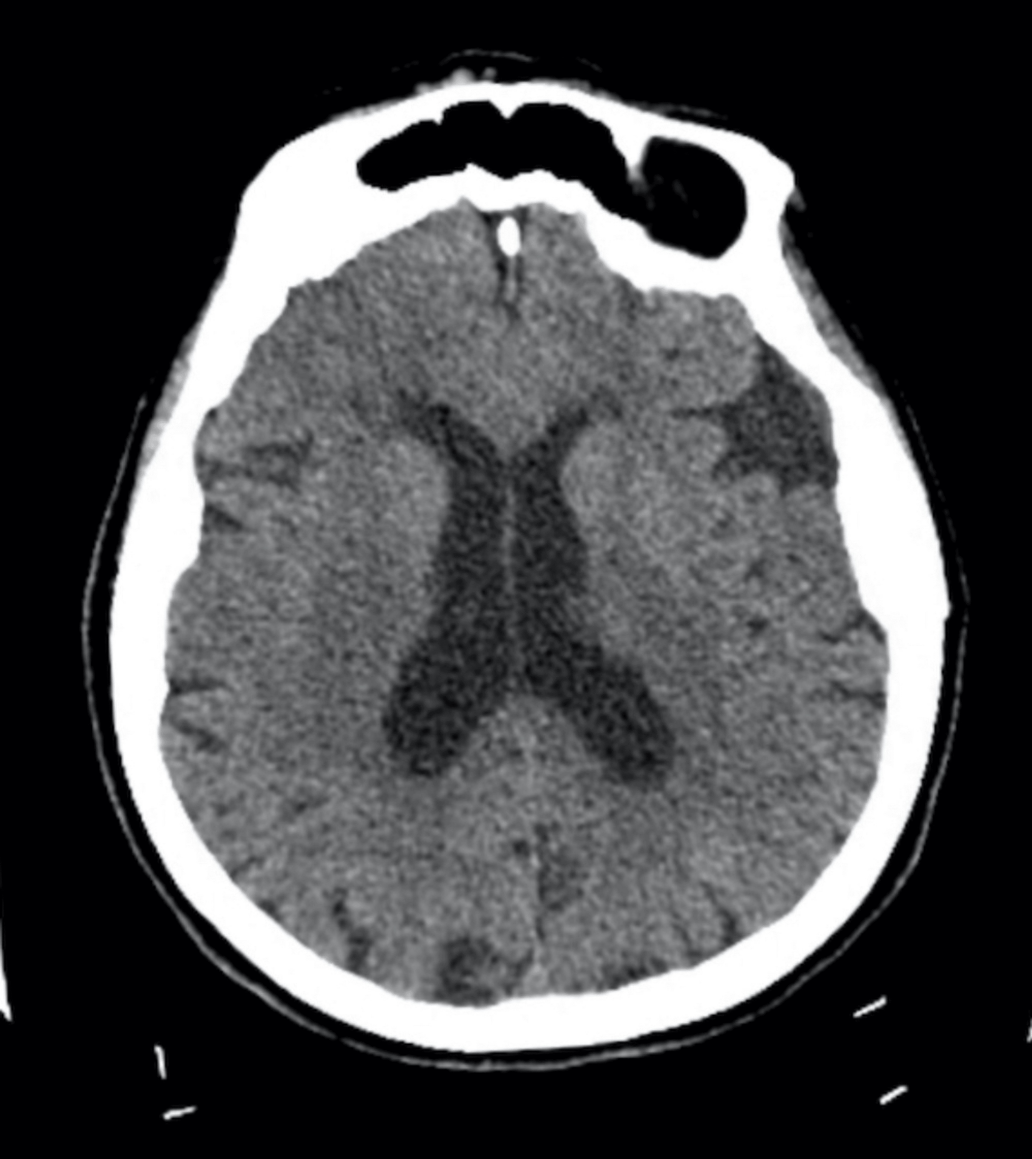
Non-contrast CT brain (axial) showing the absence of acute stroke CT: computed tomography

On physical examination in the emergency room, she had slurred speech with a stutter. The vitals and the remainder of the neurological examination were normal. The initial diagnosis was a stroke. A code stroke was initiated. The initial non-contrast head computed tomography (CT) was unremarkable for acute pathology (Figure 1). Labs did not reveal significant renal dysfunction or hepatic dysfunction (Table 1), and there were no signs of infection or notable electrolyte imbalances that could have produced stroke-like symptoms. The patient was not hypoxic.

**Table 1:** Renal and liver function tests revealed only mild abnormalities that did not explain the patient's symptoms

Blood Test	Value	Normal Range
Blood urea nitrogen (BUN)	30 mg/dL	8-23 mg/dL
Creatinine	1.17 mg/dL	0.5-1 mg/dL
Aspartate aminotransferase (AST)	35 U/L	15-37 U/L
Alanine aminotransferase (ALT)	24 U/L	10-35 U/L
Alkaline phosphatase	87 U/L	34-104 U/L
Bilirubin	0.5 mg/dL	0-1.1 mg/dL

**Figure 2:**
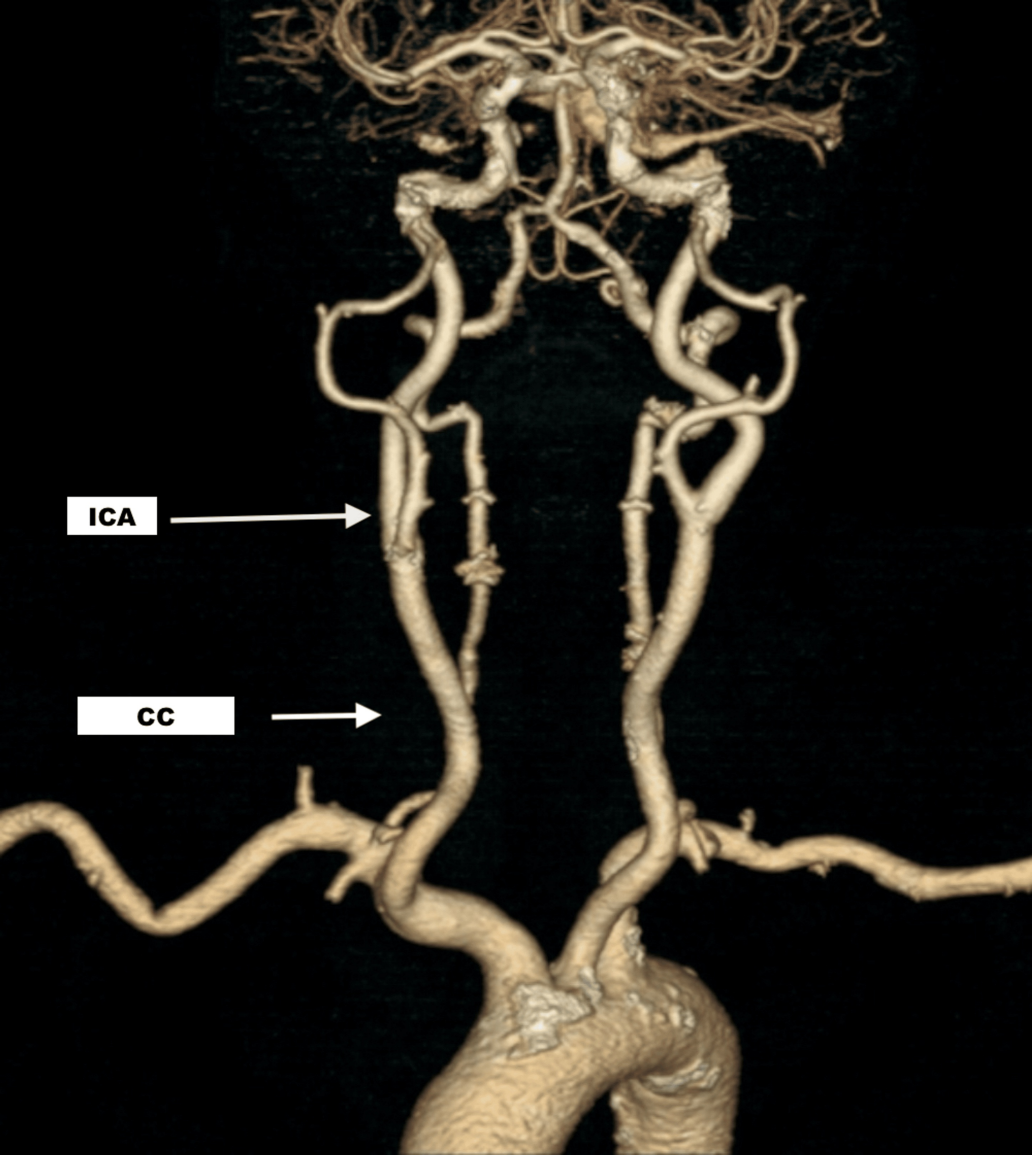
CTA neck (axial) showing the absence of significant occlusion in the common and internal carotid arteries CTA: computed tomography angiogram; ICA: internal carotid artery; CC: common carotid artery

**Figure 3:**
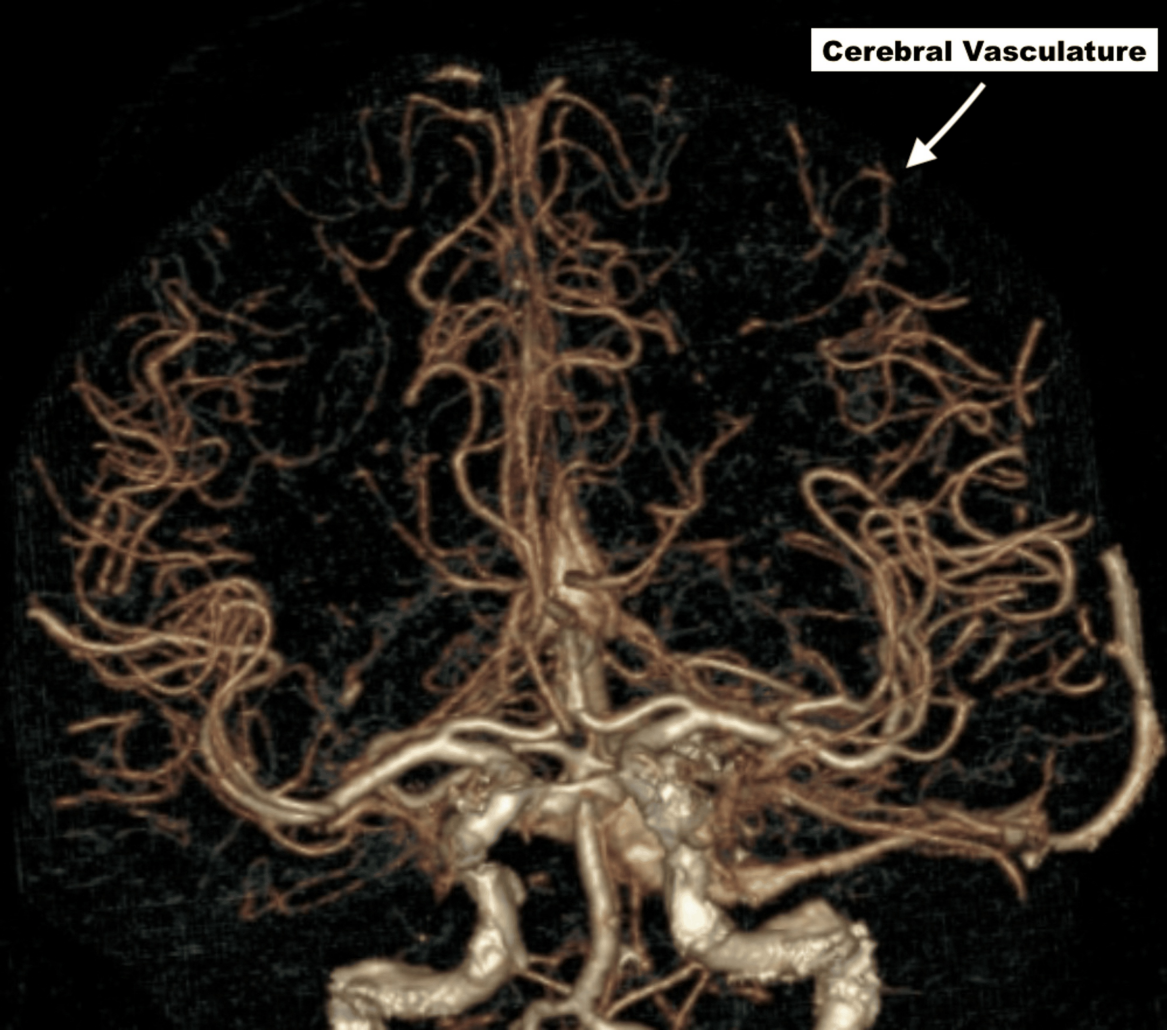
CTA brain (axial) showing absence of occlusion in the large cerebral vessels (cerebral vasculature) CTA: computed tomography angiogram

**Figure 4:**
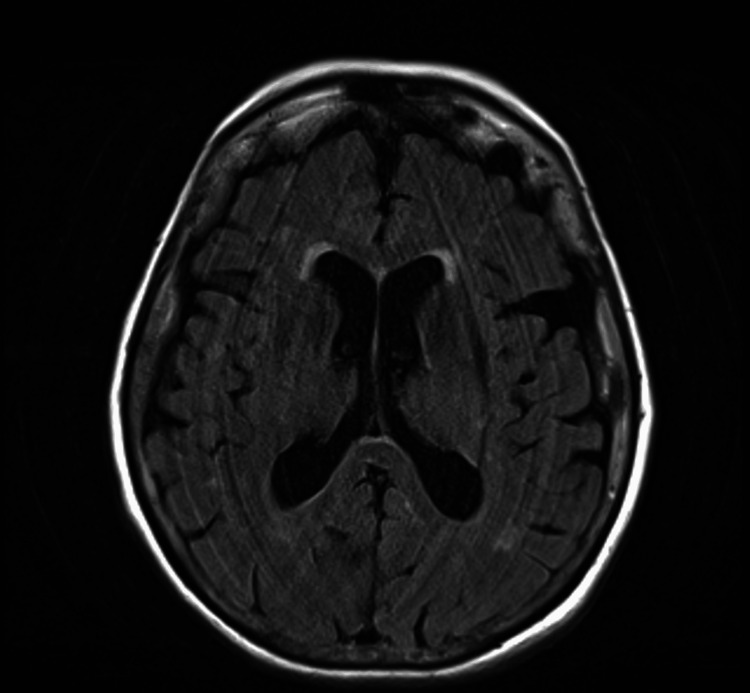
MRI brain showing absence of acute stroke MRI: magnetic resonance imaging

**Figure 5:**
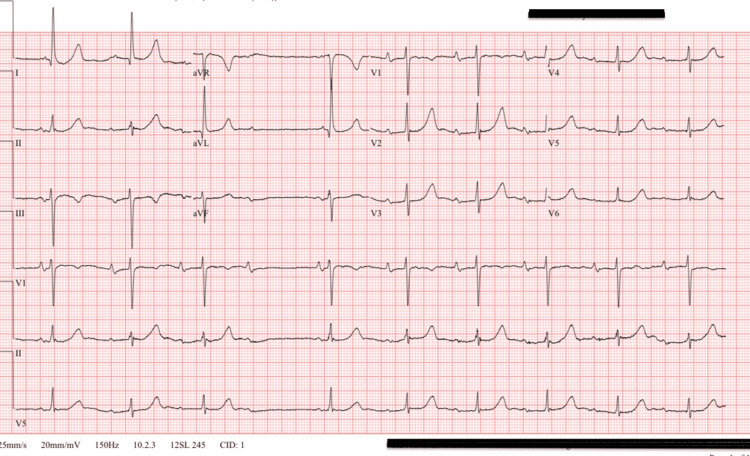
Electrocardiogram showing sinus rhythm with a Mobitz 1 type second-degree heart block

The CT angiogram (CTA) was negative for large-vessel occlusion (Figures 2-3). Magnetic resonance imaging (MRI) of the brain showed no acute infarct (Figure 4). Her electrocardiogram showed sinus rhythm with a Mobitz 1 second-degree heart block (Figure 5), which was baseline for her, and telemetry showed no arrhythmias. Her symptoms resolved spontaneously within half an hour of onset. After overnight observation, she was discharged on hospital day 2. Because imaging ruled out acute cerebrovascular pathology and clinical assessment excluded hypoxia, hypoglycemia, infection, and electrolyte imbalances, the discharge diagnosis was presumed to be a complication related to subcutaneous lidocaine administration. She returned for outpatient follow-up the following week without any residual neurological deficits.

## Discussion

The use of local anesthetics is a long-standing and common medical practice [4], and lidocaine remains one of the most frequently utilized agents due to its rapid onset and intermediate duration of action [5]. Local anesthetics exert their effects by reversibly blocking sodium channels in neuronal cell membranes, inhibiting action potential propagation and pain signal transmission [5]. Despite their routine use, local anesthetics may pose risks to patients. Local anesthetic systemic toxicity (LAST) is a rare but potentially life-threatening complication [6]. Furthermore, despite the widespread use of local anesthetics, a lack of awareness and standardized management of LAST persists in healthcare settings [6]. The relationship between local anesthetic use and neurological complications is noteworthy, especially given its widespread application, even if such events are rare [7]. Central nervous system (CNS) symptoms are often part of local anesthetic toxicity, and manifestations can include slurred speech and irrational conversation [4].

Systemic toxicity can occur if the recommended dose is exceeded, if a major vessel is inadvertently injected, or as an idiosyncratic response [5]. These symptoms can occur even within recommended dose ranges, particularly in high-risk populations such as the elderly, pregnant women, or those with advanced end-organ dysfunction [6]. Metabolic disturbances and injections in highly vascular sites may also increase the risk of toxicity [6]. 

In this case, the patient received 20 cc of 1% lidocaine subcutaneously. Although this dose is within standard limits, her advanced age and potentially altered pharmacokinetics may have contributed to transient CNS symptoms. Furthermore, injection into a highly vascular site or inadvertent intravascular administration may have increased systemic absorption. The 20 cc volume also appears larger than typically required for an ILR insertion, and a smaller dose likely would have provided adequate anesthesia.

To minimize the risks associated with local anesthetic use, clinicians should adhere to the following guidelines [6,8]: use the lowest effective dose (calculated based on lean body weight), perform slow and incremental injections, aspirate prior to injection to avoid intravascular delivery, and identify patients with higher susceptibility to local anesthetic toxicity.

Transient neurological, stroke-like symptoms, as seen in this case, appear to be self-limiting but warrant diagnostic evaluation to rule out acute cerebrovascular events. Appropriate imaging (e.g., CT, MRI) and laboratory workup may be essential to exclude alternative etiologies [4].

## Conclusions

This report describes a case of transient neurological complications following subcutaneous lidocaine administration. Imaging ruled out acute cerebrovascular pathology, and clinical evaluation excluded hypoxia, hypoglycemia, infection, and electrolyte imbalances, supporting the conclusion that her symptoms were likely secondary to the local anesthetic. This case highlights a rare but important potential complication. It also outlines the workup that followed the development of these complications. 

Given the widespread use of lidocaine as a local anesthetic, particularly in outpatient settings, clinicians should remain aware of the possibility of neurological side effects and the risk factors that may predispose patients to LAST even when standard doses are used. Safe dosing, proper injection technique, minimizing anesthetic volume, and recognizing high-risk patients, such as those of advanced age, are essential steps in preventing such adverse events.
